# Optic Nerve Sheath Diameter Ultrasound Evaluation in Intensive Care Unit: Possible Role and Clinical Aspects in Neurological Critical Patients' Daily Monitoring

**DOI:** 10.1155/2017/1621428

**Published:** 2017-03-21

**Authors:** M. Toscano, G. Spadetta, P. Pulitano, M. Rocco, V. Di Piero, O. Mecarelli, E. Vicenzini

**Affiliations:** ^1^Department of Neurology and Psychiatry, Sapienza University of Rome, Rome, Italy; ^2^Policlinico Umberto I, Rome, Italy; ^3^Department of Medical and Surgical Science and Translational Medicine, Sapienza University of Rome, Rome, Italy

## Abstract

*Background*. The increase of the optic nerve sheath diameter (ONSD) is a reliable, noninvasive sonographic marker of intracranial hypertension. Aim of the study was to demonstrate the efficacy of ONSD evaluation, when monitoring neurocritical patients, to early identify malignant intracranial hypertension in patients with brain death (BD).* Methods*. Data from ultrasound ONSD evaluation have been retrospectively analyzed in 21 sedated critical patients with neurological diseases who, during their clinical course, developed BD. 31 nonneurological controls were used for standard ONSD reference.* Results*. Patients with neurological diseases, before BD, showed higher ONSD values than control group (CTRL: RT 0.45 ± 0.03 cm; LT 0.45 ± 0.02 cm; pre-BD: RT 0.54 ± 0.02 cm; LT 0.55 ± 0.02 cm; *p* < 0.000) even without intracranial hypertension, evaluated with invasive monitoring. ONSD was further significantly markedly increased in respect to the pre-BD evaluation in neurocritical patients after BD, with mean values above 0.7 cm (RT 0.7 ± 0.02 cm; LT 0.71 ± 0.02 cm; *p* < 0.000), with a corresponding dramatic raise in intracranial pressure. Logistic regression analysis showed a strong correlation between ONSD and ICP (*R* 0,895, *p* < 0.001).* Conclusions*. ONSD is a reliable marker of intracranial hypertension, easy to be performed with a minimal training. Routine ONSD daily monitoring could be of help in Intensive Care Units when invasive intracranial pressure monitoring is not available, to early recognize intracranial hypertension and to suspect BD in neurocritical patients.

## 1. Introduction

Since the last few years, eye sonography detection of an increased optic nerve sheath diameter (ONSD) has been considered a reliable noninvasive indicator of intracranial hypertension [1–14]. Intracranial pressure (ICP) is a fundamental parameter for monitoring the neurocritical patients since, when it overrides mean arterial blood pressure, cerebral perfusion stops and BD occurs after cerebral circulatory arrest [15]. For these reasons, authors suggest a strict ICP monitoring, even through invasive methods, in the neurological critical patients in Intensive Care Units (ICUs) [16], to promptly treat those who develop intracranial hypertension. Nonetheless, the risk/benefit of invasive ICP monitoring has to be evaluated, considering that it requires specific personnel for the catheter insertion, maintenance, and troubleshooting, and it has been associated with cerebral damage and infective risks. Thus, since all these factors must be kept in mind when deciding whether to use invasive ICP monitoring, to date there are no sufficient evidences to suggest the routine use of ICP invasive monitoring [16].

On the other hand, in neurocritical, sedated, and mechanically ventilated patients, BD has to be recognized as soon as possible, in order to identify the potential candidates to be selected for organ donation, thus minimizing the time that could lead to organs deterioration. Clinically, BD diagnosis is suspected in case of unresponsive coma, persistent apnea, and absence of brainstem reflexes, all signs that are underdiagnosed during sedation. Moreover, these patients are usually already admitted to ICUs since days before BD occurrence, and their clinical neurological state is continuously clinically monitored to early detect these signs of deterioration, being easier to be suspected when a dramatic increase of ICP is also observed. When BD is suspected, a clinical and instrumental diagnostic protocol is then soon started. The diagnostic protocol, slightly different in each country, is usually composed of a multidisciplinary approach; beyond clinical and respiratory parameters evaluation, it usually includes an EEG, showing the absence of cortical electrical activity, as well as several “ancillary tests” (i.e., somatosensory evoked potentials and, in specific cases, blood flow evaluation with Transcranial Doppler, cerebral angiography, and other conventional radiological imaging) [17]. This multidisciplinary approach is strictly conservative and guarantees the highest sensibility and specificity for final BD diagnosis. However, it requires different specialists “on call,” to be ready when their cooperation is needed; thus, the task of coordinating may be difficult in every day ICU practice, further lengthening the times to final BD declaration [18, 19] with the possibility of organ deterioration in case of consent to harvest, a subject of actual social relevance.

Ultrasound devices are nowadays widely available in ICUs, but while Transcranial Doppler sonography for the identification of cerebral circulatory arrest requires trained and skilled sonographers, ONSD evaluation, to early detect the optic nerve sheath swelling in cases of intracranial hypertension, may be performed with minimal training and, above all, noninvasively [20, 21].

The aim of the study was to demonstrate the efficacy and simplicity of ONSD ultrasound evaluation in ICU in neurocritical patients monitoring, in order to early identify malignant intracranial hypertension in patients with BD.

## 2. Materials and Methods

### 2.1. Subjects

Neurocritical patients, admitted to ICU for acute neurological diseases (intracerebral hemorrhage, subarachnoid hemorrhage, trauma, tumor, and postanoxic coma) from September 2015 to April 2016, were included in this study.

Data from ultrasound ONSD evaluation have been retrospectively analyzed in those patients, sedated and mechanically ventilated, who subsequently developed BD during their clinical course. In all BD patients, BD was diagnosed according to the following criteria laid down by Italian law: (1) unresponsiveness and absence of brainstem reflexes, evaluated by means of a neurological examination; (2) absence of spontaneous breathing and apnea test revealing PCO2 > 60 mmHg and pH < 7.40; (3) absence of cortical electrical activity on the EEG tracing recorded at high sensitivity (2 *μ*V/mm), in the basal condition and under acoustic and pain stimulation, for at least 30 minutes at the beginning and at the end of the 6-hour evaluating protocol [19].

No other confirmatory tests (such as somatosensory evoked potentials) were used.

Right and Left ONSDs measured daily before BD occurrence (pre-BD) and before the ending of BD declaration protocol (post-BD) were obtained. In these patients, ICP invasive monitoring was retrospectively analyzed as well.

To obtain intraobserver normal reference values, patients without central nervous system damage or clinical signs of intracranial hypertension were used as control group.

Patients received the best medical treatment and the study involved usual standard monitoring measures used in ICUs. Written informed consent was obtained from control group patients. The study was approved by the local ethics committee.

### 2.2. ONSD Ultrasound Evaluation

In both patients and control, ONSD was evaluated with ultrasound, as already described in literature [1–14], with a 12 MHz linear ultrasound probe with reduced acoustic power [mechanical index (MI): 0.2]. The probe was adjusted to give a suitable angle for displaying the entry of the optic nerve into the globe and measurement performed at the depth of 3 mm behind the ocular globe. Right and left ONSD were measured in the transversal plane, with slight rotation of the probe to obtain the better optic nerve visualization.

### 2.3. Statistical Analysis

Normality of the distributions was assessed with the Shapiro-Wilk Normality Test; according to the result of normality analysis, Student's *t*-test or Wilcoxon test were used to compare mean ONSD values differences. ANOVA with post hoc analysis with Bonferroni and Scheffé corrections were used to determine interactions between groups. Correlation between ONSD and ICP was assessed by means of logistic regression analysis.

All tests were two-tailed with significance set to alpha = 0.05. Data were analyzed with SPSS software for Windows (Version 18.0).

Based on ONSD normative values reported in the literature [11], a priori power analysis was performed by means of Wilcoxon-Mann–Whitney tests (a priori computed required sample size). We calculated that a total sample of 20 neurological critical patients would provide a power of 95% to reach a statistical significance in ONSD values differences between pre-BD and controls (*α*-error 0.05, effect size 1.21) and between pre-BD and post-BD as well (*α*-error 0.05, effect size w 1.26).

Post hoc power analysis (Wilcoxon-Mann–Whitney tests, post hoc computed achieved power) was used to determinate the level of statistical power achieved for statics validation.

## 3. Results

We retrospectively analyzed data from 21 neurocritical patients (M/F 11/10, mean age: 54 ± 13.5), sedated and mechanically ventilated, who subsequently developed BD during their clinical course. Out of these, two were submitted to decompressive craniectomy.

As control group, we also enrolled 31 patients (M/F 14/17, mean age: 56.3 ± 10.3) without central nervous system damage or clinical signs of intracranial hypertension.

In the control group, mean ONSD was 0.45 ± 0.03 cm for right side and 0.45 ± 0.02 cm for left side. No differences for ONSD side were observed. In the neurological patients, sedated and mechanically ventilated, who subsequently developed BD, ONSD values before BD occurrence were slightly significantly higher than in the control group (pre-BD: RT 0.54 ± 0.02 cm; LT 0.55 ± 0.02 cm; CNTR: RT 0.45 ± 0.03 cm; LT 0.45 ± 0.02 cm; *p* < 0.000). At this stage, as expected, intracranial invasive pressure measurement in neurocritical patients was within normal values (7 ± 2 mmHg).

**Figure 1:**
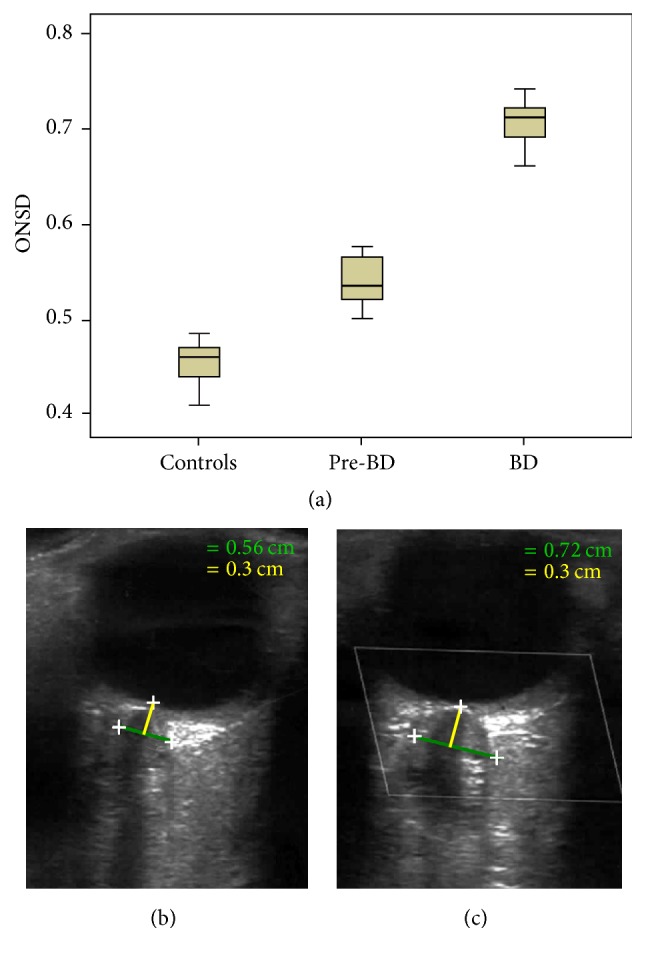
Box plot with interquartile range (IQR) distribution of ONSD values in patients and controls (a), with an example of two subsequent ONSD measurements in a patient in the pre-BD phase (b) and after BD occurrence (c). Marks indicate the optic nerve sheath, green line indicates the diameter, and yellow line indicates the measurement performed at the depth 0.3 cm behind the ocular globe.

After BD occurrence, ONSD was further significantly markedly increased, greater than 0.7 cm (RT 0.7 ± 0.02 cm; LT 0.71 ± 0.02 cm; *p* < 0.000) in respect to the pre-BD phase, except for the 2 patients who had submitted to decompressive craniectomy, in whom ONSD was substantially unchanged (before BD: RT 0.56 ± 0.01 cm; LT 0.57 ± 0.01 cm. After BD: RT 0.56 ± 0.03; LT 0.58 ± 0.04 cm). The increase of ONSD was constantly correlated with elevated values of ICP (38 ± 9 mmHg) in the patients with BD who were not submitted to decompressive surgery.  Figure 1 shows box plot with interquartile range (IQR) distribution of ONSD values in patients and controls (a), with an example of two subsequent ONSD measurements in a patient in the pre-BD phase (b) and after BD occurrence (c). Grouped and individual data are reported in Tables 1 and 2, respectively.

ANOVA showed a significant difference between the three groups (CTRL, pre-BD, and post-BD. RT ONSD: *F* = 477.2; *p* < 0.000. LT ONSD: *F* = 610.4; *p* < 0.000) confirming the differences observed within the groups at multiple comparisons after Bonferroni and Scheffé corrections.

**Figure 2:**
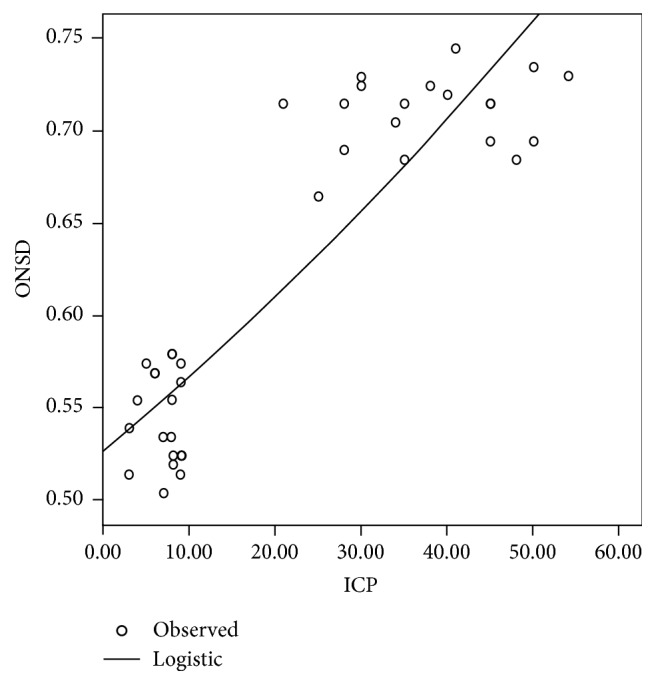
Logistic regression analysis (ONSD versus ICP). See the text for the details.

Logistic regression analysis showed a strong correlation between ONSD and ICP (*R* 0,895, *p* < 0.001). Correlation graph was reported in Figure 2.

Post hoc power analysis (Wilcoxon-Mann–Whitney tests, post hoc computed achieved power) showed that a sample size of 21 neurological critical patients and 31 controls provided sufficient power for statics validation (pre-BD versus control: power 1; *α* err 0.05; effect size w 4.03; pre-BD versus post-BD: power 1; *α* err 0.05; effect size w 4.91).

## 4. Discussion

From a pathophysiological point of view, BD is caused by an acute central nervous system damage, which may be related to a direct “primary” lesion, such as intracerebral bleeding, severe cerebral concussion, and brain tumors, or to indirect “secondary” causes such a diffuse prolonged cerebral hypoxia following cardiopulmonary resuscitation. The final consequence of all these conditions is that they determine a dramatic brain edema and cerebral parenchyma swelling with uncontrollable intracranial hypertension, leading to cerebral circulatory arrest and consequent cessation of brain electrical activity [15].

Several clinical studies described the increase of ONSD evaluated with ultrasound as a reliable noninvasive method to detect intracranial hypertension in neurosurgery and ICUs [1–14]. As a matter of fact, the optic nerve sheath is directly connected with the subarachnoid space, and, differently from the skull that it is inextensible, the intraorbital subarachnoid meningeal prolongation is then free of swelling with the pressure increase in the cerebrospinal fluid. Historically, the sign of the papilledema is a typical expression of this phenomenon.

The evaluation of the ONSD with ultrasound seems then to be a reliable indicator of intracranial hypertension, with high intra- and interobserver reliability (with up to 2 decimals of centimeter) and with a whole range from 0.43 to 0.76 mm [2]. A growth of the sheath diameter from 0.4 to 0.45 cm is detectable among first 4 years of life [10, 22], while normal adults have mean ONSD values of about 0.5 cm [23]. In neurological patients with stroke, intracerebral or subarachnoid hemorrhage, wider values, of about 0.59 to 0.63 cm, are reported [24]; otherwise, there are no reported values lower than 0.58 cm when ICP is detected over 20 mmHg [3]. Only one study reports an increase of ONSD of up to 0.72 cm in patients with BD, but without available data on ICP monitoring [7].

Our data are in line with all these findings, confirming the ONSD values in the control group, and showing a slight wider optic nerve sheath diameter in neurological critical patients, ranging from 0.50 to 0.58 cm, and with normal values of ICP (i.e., lower than 10 mmHg).

The main finding of our study is that we have observed the widest ONSD values occurring after BD, from 0.68 to 0.75 cm, and with ICP values from 28 to 54 mmHg. Logistic regression analysis confirmed this strong correlation between increased ONSD and raise in ICP, with mean OSND values above 0,68 cm in BD patients (Figure 2, patients lying on the upper side of the graph) reflecting a raise in intracerebral pressure higher than the mean arterial blood pressure. Such optic nerve sheath swelling could then be considered the expression of the final, extreme compensative strategy to the massive brain swelling in the inextensible skull, and correlated with the demonstration of the malignant ICP increase at the time of BD diagnosis.

Moreover, the confirmation that intracranial pressure fatally affects ONSD is indirectly demonstrated by the lack of ONSD increase in our patients with decompressive craniectomy; the lack of ONSD swelling in these patients could be explained by incapacity of the intracranial pressure to reach such a dramatic increase as to induce cerebral circulatory arrest or to determine optic sheath swelling over 0.7 cm. In other words, since craniectomy prevents malignant ICP increase, ONSD normal values in the two craniectomies are actually the proof of concept that OSND measurement is strongly related to ICP.

A limitation of the present study is a missing control group consisting in ICU patients who finally did not develop BD. Nonetheless, in patients who do not develop BD, no suspicion of fatal intracranial hypertension leading to circulatory arrest, brain swelling, and increase of ONSD may be hypothesized. As a matter of fact, we compared ONSD in the same patient before and after BD developing; given the small sample of patients, this otherwise helped to avoid any intersubject factors known to influence ONSD measurement, thereby resulting in a more reliable representation of how ONSD values change according to malignant intracranial hypertension.

The importance of our findings that represent our final take-home message is not the role of a single ONSD measurement or the absolute value of ONSD for BD diagnosis. It is indeed pleonastic that BD diagnosis in ICU is not made upon the only evaluation of ONSD. In fact, even though ONSD is significantly greater in subjects with BD, quantification of ONSD cannot discriminate BD subjects from comatose ones with raised ICP with 100% certainty [25].

Otherwise, it seems noteworthy to underline that ONSD ultrasound evaluation could be of strong help during clinical monitoring to early detect the cases with raised intracranial pressure and to suspect clinical deterioration, when dramatic ONSD increase, over 0.7 cm, is observed over time. This is particularly true in neurocritical patients sedated and mechanically ventilated or when invasive ICP monitoring is not available so that, in these patients, further investigations for BD diagnosis can be started with more consistent data and avoiding useless evaluations.

Moreover, ONSD evaluation with ultrasound can be performed with short training from almost every medical and paramedical personnel. Thus, ONSD appears as an easy and noninvasive diagnostic tool to early identify malignant intracranial hypertension during anesthesiological routine daily follow-up of critical patients.

## 5. Conclusion

ONSD evaluation with ultrasound may be easily performed, with minimal training, also by nonspecific neurosonologist. ONSD routine monitoring may be of help in Intensive Care Units, to early detect patients with raised intracranial pressure when invasive ICP monitoring is not available; it also allows to suspect BD when ONSD is rapidly increased over time and over 0.7 cm, especially in those neurocritical patients, sedated and mechanically ventilated, who develop signs of cerebral deterioration.

## Figures and Tables

**Table 1:** ONSD mean values (cm ± SD) in controls (CTRL), in neurological patients before brain death (pre-BD), after brain death (after BD) and in the 2 patients with brain death and decompressive craniectomy. Intracranial pressure values are also reported (ICP, mmHg).

	CTRL (*n* = 31)	Pre-BD (*n* = 21)	ICP	After BD (*n* = 19)	ICP	BD + craniectomy (*n* = 2)
Right	0.45 ± 0.03	0.54 ± 0.02	7 ± 2	0.7 ± 0.02	38 ± 9.4	0.56 ± 0.03
Left	0.45 ± 0.02	0.55 ± 0.02		0.71 ± 0.02		0.58 ± 0.04

**Table 2:** ONSD results, right (RT, cm) and left (LT, cm), and intracranial pressure (ICP, mmHg) in controls and neurological patients with brain death.

Controls			Neurological patients with outcome in brain death						
				Before brain death			After brain death		
	RT	LT	Diagnosis	ICP	RT	LT	ICP	RT	LT
Pt 1	0.48	0.47	ICH	8	0.54	0.53	45	0.71	0.72
Pt 2	0.49	0.49	ICH	7	0.52	0.55	48	0.68	0.69
Pt 3	0.44	0.43	Trauma	9	0.56	0.57	34	0.7	0.71
Pt 4	0.45	0.46	ICH	9	0.51	0.52	38	0.72	0.73
Pt 5	0.41	0.42	Trauma	8	0.58	0.58	30	0.71	0.74
Pt 6	0.48	0.47	SAH	6	0.57	0.57	21	0.72	0.71
Pt 7	0.48	0.47	Trauma	5	0.58	0.57	25	0.69	0.64
Pt 8	0.45	0.46	ICH	8	0.52	0.52	28	0.71	0.72
Pt 9	0.46	0.45	ICH	9	0.53	0.52	35	0.71	0.72
Pt 10	0.43	0.44	SAH	7	0.5	0.51	40	0.71	0.73
Pt 11	0.48	0.47	Tumor	6	0.57	0.57	45	0.68	0.71
Pt 12	0.48	0.47	Tumor	8	0.58	0.58	50	0.74	0.73
Pt 13	0.47	0.46	ICH	9	0.52	0.53	28	0.69	0.69
Pt 14	0.46	0.47	ICH	8	0.53	0.52	30	0.72	0.74
Pt 15	0.42	0.43	Trauma	4	0.54	0.57	35	0.68	0.69
Pt 16	0.44	0.45	Trauma	3	0.53	0.55	50	0.68	0.71
Pt 17	0.46	0.47	ICH	3	0.5	0.53	54	0.74	0.72
Pt 18	0.47	0.48	Postanoxic	9	0.58	0.57	41	0.75	0.74
Pt 19	0.44	0.45	Postanoxic	8	0.56	0.55	45	0.72	0.71
Pt 20	0.48	0.49							
Pt 21	0.49	0.48	Trauma + DC	/	0.57	0.58	/	0.59	0.61
Pt 22	0.48	0.47	ICH + DC	/	0.55	0.56	/	0.54	0.55
Pt 23	0.42	0.43							
Pt 24	0.42	0.43							
Pt 25	0.49	0.48							
Pt 26	0.38	0.37							
Pt 27	0.42	0.44							
Pt 28	0.39	0.4							
Pt 29	0.49	0.48							
Pt 30	0.47	0.45							
Pt 31	0.49	0.47							

ICH: intracerebral hemorrhage; SAE: subarachnoid hemorrhage; DC: decompressive craniectomy.
